# Antibiotic Resistance Profiles, Molecular Mechanisms and Innovative Treatment Strategies of *Acinetobacter baumannii*

**DOI:** 10.3390/microorganisms8060935

**Published:** 2020-06-21

**Authors:** Corneliu Ovidiu Vrancianu, Irina Gheorghe, Ilda Barbu Czobor, Mariana Carmen Chifiriuc

**Affiliations:** 1 Microbiology Immunology Department, Faculty of Biology, University of Bucharest, 050095 Bucharest, Romania; ovidiu.vrancianu@yahoo.com (C.O.V.); ilda.czobor@yahoo.com (I.B.C.); carmen.chifiriuc@bio.unibuc.ro (M.C.C.); 2 The Research Institute of the University of Bucharest, 050095 Bucharest, Romania

**Keywords:** antimicrobial resistance, β-lactamases, *Acinetobacter baumannii*, antimicrobial peptide, bacteriophage, CRISPR

## Abstract

Antibiotic resistance is one of the biggest challenges for the clinical sector and industry, environment and societal development. One of the most important pathogens responsible for severe nosocomial infections is *Acinetobacter baumannii*, a Gram-negative bacterium from the Moraxellaceae family, due to its various resistance mechanisms, such as the *β*-lactamases production, efflux pumps, decreased membrane permeability and altered target site of the antibiotic. The enormous adaptive capacity of *A. baumannii* and the acquisition and transfer of antibiotic resistance determinants contribute to the ineffectiveness of most current therapeutic strategies, including last-line or combined antibiotic therapy. In this review, we will present an update of the antibiotic resistance profiles and underlying mechanisms in *A. baumannii* and the current progress in developing innovative strategies for combating multidrug-resistant *A. baumannii* (MDRAB) infections.

## 1. Introduction

The global spread of antimicrobial resistance (AMR) is one of the global challenges of the 21st century. It is estimated that by 2050, infections caused by resistant strains will lead to 300 million deaths prematurely [1]. Bacterial strains can be naturally resistant to a particular antibiotic or become resistant through the acquisition of resistance determinants [2]. Although there are multiple causes of the resistance phenomenon, it is considered that AMR is an old phenomenon, with an accelerated evolution triggered by the abusive use of antibiotics [3]. Given the extreme mobility of antibiotic resistance genes (ARGs) and their boundaryless dissemination from humans to animals/clinical to environmental reservoirs and vice versa, reducing threats is a difficult task to achieve.

In recent years, the resistance phenomenon was encountered in most common bacterial strains causing infections, associated with an increased risk of morbidity, mortality, high treatment costs and long periods of hospitalization. One of the ESKCAPE pathogens responsible for nosocomial and community-acquired infections is *Acinetobacter baumannii*, a Gram-negative, non-motile, non-fermentative and non-sporulated bacterium from the *Moraxellaceae* family [4]. *A. baumannii* is part of the *A. baumannii*–*A. calcoaceticus* complex (Acb), initially including four species: *A. calcoaceticus*, *A. baumannii*, *A. nosocomialis* and *A. pittii* [5]. Subsequently, several other species have been proposed for inclusion in this complex: *A. seifertii* [6], *A. lactucae* [7] and *Acinetobacter* species “between 1 and 3”. Species between “1 and 3” are phenotypically identical and highly similar genotypically, thus sensitive tests are needed to differentiate them [8]. The Acb complex has become one of the biggest challenges in hospitals, primarily due to its increased resistance to carbapenems and other antibiotics, with minimal treatment options. Risk factors for colonization and infection with species within the Acb complex are extended periods of hospitalization, admission to ICUs, mechanical ventilation or exposure to antimicrobial agents [9]. Of all the species in the Acb complex, *A. baumannii* is the most widespread in hospitals. Two of the many reasons for the success of MDRAB strains are the association with chronic nosocomial infections and their unique ability to survive in extreme environmental conditions. Its reputation is mainly due to its association with severe infections caused to the US military during the wars in Afghanistan and Iraq, which is why it has been called “Iraqibacter” [10]. *A. baumannii* causes various infections, including pneumonia, urinary tract infections, skin and soft tissue infections or nosocomial meningitis [11]. Due to its extended resistome and virolome, evasion of the host’s immune effectors, ability to grow in biofilms, to survive in extreme environmental conditions, and to switch to latent growth forms with a minimal metabolic rate, the treatment options are limited, rendering *A. baumannii* one of the most critical and fearful pathogens [12,13]. In this review, we will present the antibiotic resistance profiles of *A. baumannii* strains, the main mechanisms (enzymatic and non-enzymatic) of antibiotic resistance (AR), as well as an update regarding the perspectives of new therapeutic strategies efficient against MDRAB. In addition, the discussion section will present the main challenges of therapeutic strategies and the need for further studies in response to existing limitations.

## 2. AR Profiles of *A. baumannii* Strains

Studies over the past 20 years have shown that *A. baumannii* has globally emerged as a highly troublesome nosocomial pathogen revealing multidrug resistance (MDR), extensively drug–resistant and pandrug-resistant phenotypes. Several studies revealed the involvement of aminoglycoside resistance genes, methyltransferases [14] and class 2 *β*-lactamases in the genesis of the MDR phenotypes [15]. Analysis of AR of several *A. baumannii* isolates collected from different intensive care units revealed high resistance to the most commonly used antibiotics [16,17]. Several studies reported high carbapenem, aminoglycoside and colistin resistance of *A. baumannii* strains [18,19,20,21,22,23,24,25,26,27,28,29,30,31] (Table 1). The carbapenem resistance was associated with the overexpression of OXA-51 [32] and OXA-23 [33,34] class D carbapenemases as well as of metallo-β-lactamases (MBLs) [35]. The OXA-51 and OXA-40 class D β-lactamases (CHLD) were involved in ceftazidime resistance [36,37]. Several studies revealed the association of the *bla*_OXA-23_ gene with the compound transposon Tn*2006* [38] and with the insertion sequence IS*Aba1* located upstream of the *bla*_OXA-23-_ gene that increases the carbapenem resistance expression level [39,40].

Many studies highlight the involvement with a very high frequency of resistant *A. baumannii* strains in different types of infections and the need to monitor the antibiotic consumption. The enormous adaptability of resistant strains, supported by the acquisition and dissemination of resistance and virulence markers, is a global problem that requires an imperative understanding of bacterial resistance mechanisms.

## 3. Short Characterization of the Molecular Mechanisms of AR

Resistance to antibiotics is not a recent phenomenon. Since 1942, shortly after discovering and using penicillin to fight bacterial infections, several strains of *Staphylococcus* spp. with different AR levels have been reported [45]. The introduction of a high number of new antibiotics and the implicit high level of antibiotic consumption globally has led to the development of numerous bacterial mechanisms to inactivate the action of antibiotics. The abusive consumption of antibiotics in the last 30 years has determined a selective pressure favorable for the spread of the resistance phenomenon. Currently, treating bacterial infections requires increasing doses of antibiotics and prolonged hospitalization [46]. Bacterial resistance is a natural result of the interaction of microorganisms with the environmental niche. Over time, bacteria have developed an arsenal of mechanisms to ensure their survival in a hostile environment. Thus, it is considered that bacterial strains resistant to one or more antimicrobial compounds have an intrinsic resistance (IR), mediated by the resistance determinants [47]. Generally, IR is based on the production of the enzymes that can eliminate the antimicrobial compound or prevent its intracellular binding to the target site. This ability of bacteria is characterized by maintaining a level of resistance to the antimicrobial compound, even in the absence of the previous contact [48].

Bacteria use two genetic mechanisms of defense against antibiotics: mutations, which usually interfere with the mechanism of action of the compound and the acquisition of exogenous genetic material, through horizontal gene transfer (HGT) [49]. Regarding the acquisition of exogenous material, bacteria can acquire and disseminate genes with an essential role in the spread of AR, through mobile genetic elements (MGE) [47]. The main mechanisms of AR are enzymatic (*β*-lactamases production) and non-enzymatic (alteration of membrane permeability, activation of efflux pumps and alteration of the target site).

### 3.1. Enzymatic Mechanisms

#### *β*-lactamases

*β*-Lactamases are versatile enzymes revealed by bacteria from different sources with a limited range of molecular structures. The characteristic feature of these enzymes is the ability to hydrolyze chemical compounds with a *β*-lactam ring, thus inactivating the antibacterial compound [47]. Although first described by Abraham and Chain in 1940 [50], molecular phylogenetic analyses suggest that *β*-lactamases are ancestral enzymes dating back about two billion years [51]. In contrast, plasmids encoding *β*-lactamases appeared millions of years ago [52]. β-Lactams antibiotics act by acetylating a serine site in the structure of penicillin-binding proteins (PBPs) [53]. In Gram-positive bacteria, the primary mechanism of *β*-lactam resistance is the alteration of PBP affinity for these antibiotics, while maintaining physiological functions [54]. The strategy of low-affinity mutant PBP synthesis is a primary mechanism of resistance [55]. In Gram-negative bacteria, β-lactamase synthesis is the main mechanism of resistance to *β*-lactam antibiotics. The first enzyme with *β*-lactamase activity was discovered in *Bacillus coli* by Abraham and Chain in 1940, considered today as the chromosomally encoded cephalosporinase in *Escherichia coli*. Subsequently, the synthesis of *β*-lactamases as a mechanism of resistance in Gram-negative bacteria became very common, with the discovery of several chromosomal-encoded inducible enteric β-lactamase-producing bacteria [56]. The prevalence of *β*-lactamase synthesis in Gram-negative bacteria has been favored by the occurrence of transferable plasmids encoding a wide range of enzymes involved in the spread of *β*-lactam resistance. *β*-lactamases were classified based on molecular [57] and functional [58,59] structure analysis. Ambler grouped the *β*-lactamases into four classes (A, B, C and D), depending on the amino acid sequences. For classes A, C and D, the active enzyme site contains serine and class B includes Zn-dependent metallo-enzymes [57]. In the functional classification of Bush, Jacoby and Medeiros, *β*-lactamases are divided into three groups, depending on the degraded β-lactam substrate and the effects of inhibitors. The first group includes class C cephalosporinases from the molecular structure classification. The second group comprises *β*-lactamases other than those from the first group, which have serine at the active site. The third group includes MBLs corresponding to class B of Ambler’s classification. All four *β*-lactamases Ambler classes have been identified in *A. baumannii* strains.

Class A β-lactamases are the most frequent cause of *β*-lactam resistance [60]. These enzymes are inhibited by clavulanate and can hydrolyze penicillins and cephalosporins more efficiently than carbapenems [61]. Phenotypic and biochemical analyses have led to the identification of several functional groups of class A *β*-lactamases, currently known over 2000, mostly identified in Gram-negative bacilli [62]. Functional types of class A *β*-lactamases have different molecular variants that demonstrate their ability to hydrolyze cephalosporins and carbapenems [62,63]. In *A. baumannii*, many class A *β*-lactamases such as TEM, GES, CTX-M, SHV, SCO, PER, CARB, VEB or KPC were found (Table 2). Of these, most are broad-spectrum *β*-lactamases (ESBL) (SHV-5, PER-1, PER-2, PER-7, TEM-92, CTX-M-15, VEB-1, GES-14, CARB -10, CTX-M-2) and some are narrow-spectrum (TEM-1, SCO-1).

In contrast to class A enzymes in which the enzyme active site contains serine, class B β-lactamases or MBLs have Zn or another heavy metal in the catalytic site [64]. MBLs are part of the third group of functional classification developed by Bush and Jacoby and have the ability to hydrolyze almost all β-lactam antibiotics except monobactams [61]. Because of the metal from the active enzyme site, the enzymatic activity of these types of β-lactamases can be inhibited by chelating agents such as ethylenediaminetetraacetic acid (EDTA) [65]. There have been reported several MBLs, such as imipenemases (IMPs) [66], Verona integron-encoded MBL (VIM) [67], Sao Paolo MBL (SPM) [68], imipenemase from Germany (GIM) [69], MBL from New Delhi (NDM) [70], Seoul imipenemase (SIM), Australian imipenemase (AIM) and imipenemase from Florence (FIM) [71]. In *A. baumannii*, a wide range of MBLs has been identified (Table 2).

Class C β-lactamases are encoded by the *ampC* gene found in *Enterobacteriaceae* and functionally is a non-inducible cephalosporinase framed by Bush and Jacoby into group 1 [72,73,74]. These *β*-lactamases may confer resistance to cephamycins, penicillins or cephalosporins [61]. Investigation of 105 MDRAB strains from China demonstrated the presence of the *bla*_ampC_ gene in 65 strains [75]. An analysis of 23 *A. baumannii* strains from Taiwan revealed the presence of *ampC*-type *β*-lactamases in all strains [76].

Class D β-lactamases or CHLD also called oxacillinases (OXA) due to their ability to hydrolyze oxacillin, have serine in the active catalytic site and are included in functional group two of the Bush and Jacoby classification [59]. Over 400 OXA enzymes have been characterized, mostly having the ability to hydrolyze carbapenems. In *A. baumannii*, the presence of OXA-type *β*-lactamases which hydrolyze carbapenems is one of the significant mechanisms of resistance [77,78]. OXA enzymes such as OXA-23, OXA-24/40, OXA-58, OXA-143 and OXA-235 are among the most prevalent in *A. baumannii* strains (Table 2). OXA-23 was identified in Scotland [79], later disseminated globally, now reaching a high frequency in *A. baumannii* isolates [38,80,81,82]. Genes encoding OXA-type *β*-lactamases have been identified mainly chromosomally or plasmid located in *A. baumannii* strains [83,84].

**Table 2:** Main β–lactamases in *A. baumannii* strains.

Class/Group	Enzyme	Location	References
Class A *β*-lactamases	CTX-M (-2, -15, -43)	C, P ^a^	[85,86,87,88,89,90,91]
	TEM (-1, -92, -116)	P	[92,93,94,95,96,97,98]
	GES (-1, -5, -11, -14, -15)	P	[99,100,101,102]
	PER (-1, -2, -3, -7)	C, P	[103,104,105,106]
	VEB (-1, -3, -7)	C	[104,107]
	KPC (-2, -3, -10)	–	[108,109,110]
	SCO-1	P	[111]
	CARB (-4, -10)	C, P	[112,113]
	SHV (-5, -12)	C	[114,115]
Class B *β*-lactamases	IMP (-1, -2, -4, -5, -6, -8, -10, -11, -14, -19, -24)	I	[116,117,118,119,120,121,122,123,124]
	VIM (-1, -2, -3, -4, -6, -11)	I	[121,125,126]
	NDM (-1, -2, -3)	C, P	[127,128,129,130]
	SIM-1	I	[131]
	SPM-1	P	[68]
	GIM-1	I, P	[69]
	FIM-1	C	[71]
Class C *β*-lactamases	AmpC	C	[132,133,134]
Class D β-lactamases	OXA-23-like (-23, -27, -49, -73, -102, -103, -105, -133, -134, -146, -165- OXA-171, -225, -239)	C, P	[35,135,136,137,138]
	OXA-40/24-like (-40, -25, -26, -72, -139, -160, -207)	C, P	[138,139,140]
	OXA-51-like (-51, OXA-64– OXA-71, OXA-75– OXA-80, OXA-82- OXA-84, OXA-86– OXA-95, OXA-98– OXA-100, -104, OXA-106– OXA-113, OXA-115– OXA-117, OXA-120– OXA-128, OXA-130– OXA-132, -138, -144, OXA-148– OXA-150, OXA-172– OXA-180, OXA-194– OXA-197, OXA-200– OXA-203, -206, -208, -216, -217, -219, -223, -241, -242, OXA-248– OXA-250, -254)	C, P	[32,141,142,143,144,145,146]
	OXA-58-like (-58, -96, -97, -164)	C, P	[122,147,148,149]
	OXA-143-like (-143, -182, -231, -253, -255	P	[150,151,152,153]
	OXA-48-like (-48, -48b, -162, -163, -181, -199, -204, -232, -244, -245, -247).	C, P	[154,155,156,157,158]
	OXA-235	C, P	[159]

^a^ C—chromosomally; P—plasmid; I—integron; “–”—unknown.

Aminoglycosides (AGs) are among the most important antibiotic classes used to treat nosocomial infections caused by *A. baumannii* strains [160]. Enzymatic modification of AGs through production of aminoglycoside-modifying enzymes (AMEs) is the main mechanism of resistance in *A. baumannii*. Depending on the mechanism by which it acts, AMEs are classified into acetyltransferases, phosphotransferases and nucleotidyl transferases [161,162]. The main AMEs involved in AGR in *A. baumannii* are *aac (3′)-I*, *aph (3′)-I*, *aph (3′)-VI*, *aac (6′)-Ib*, *ant (2″)-Ia, ant (3′)–I*, *aac(3)-Ia [aacC1], aac(3)-IIa [aacC2], aac(6′)-Ib [aacA4], aac(6′)-Ih, aac(6′)-Im, aph(3′)-Ia [aphA1], aph(3′)*-VIa *[aphA6], ant(3″)Ia [aadA1]* and *ant(2″)-Ia [aadB]*, aac(6′)-I ad, aac(6′)-II and ant(4′)-I [163,164,165,166,167,168,169,170,171,172,173]. Genes encoding AMEs can be transferred as part of gene cassettes in the case of integrons, as well as through conjugation mechanisms [174]. Other resistance mechanisms are 16S ribosomal methylases [175,176]. Methyltransferases produce changes at A1408 and G1405 of the small 16S ribosomal unit, which are considered essential for the interaction of the antibiotic with the ribosome and lead to AR [177].

### 3.2. Non-Enzymatic Mechanisms

#### 3.2.1. Activation of the Efflux Pumps

*A. baumannii* efflux systems are encoded by chromosomal genes responsible for resistance to several antimicrobial agents in case of overexpression [178]. Genes encoding the efflux mechanisms of specific agents are usually found in MGEs (transposons, integrons, plasmids), their acquisition from other organisms contributing to resistance. MDR efflux systems are generally encoded by chromosomal genes that are expressed constitutively, contributing to IR or expressed following mutation and leading to acquired resistance [179,180].

In *A. baumannii*, four categories of efflux pumps were identified: RND superfamily (resistance–nodulation–division superfamily), MATE (multidrug and toxic compound extrusion family), MFS (major facilitator superfamily) and SMR (small multidrug resistance transporters) [77]. Of the four efflux systems, the RND system is more represented in *A. baumannii*, which includes the AdeABC pump, with an essential role in resistance to antimicrobial agents, especially aminoglycosides. The AdeABC pump consists of three components: the inner membrane of the pump (AdeA), the major AdeB fusion protein and the outer membrane factor (AdeC) [181]. AdeABC is encoded by the *adeRS* operon, whose expression occurs when the efflux pump is exposed to an excessive concentration of toxic agents or antibiotics, leading to an MDR phenotype [182]. Overexpression of the adeABC efflux system is caused either by insertion of the IS*Aba1* element upstream of the adeABC operon or by punctiform mutations in the *adeR* and *adeS* genes [183]. Point mutations in the *adeRS* operon regulate AdeABC activity, causing resistance to several antibiotics such as aminoglycosides, β-lactams, fluoroquinolones, tetracyclines, macrolides and chloramphenicol [175,184]. The adeRS operon is also involved in *A. baumannii* biofilm formation. Inactivation of the *adeRS* operon inhibits the biofilm formation due to decreased expression of the AdeABC efflux pump [185]. The RND family also includes the AdeFGH and AdeIJK efflux pumps, which are associated with tigecycline resistance [183] and the transcriptional regulators AdeL and AdeN control their expression [186,187].

Another category of efflux pumps encountered in *A. baumannii* is MATE. This family includes the AbeM efflux pump, characterized by Su et al. They reported the involvement of AbeM, along with other efflux pumps, in resistance to norfloxacin, ofloxacin, ciprofloxacin, gentamicin, doxorubicin, triclosan and imipenem [188,189,190].

The MFS superfamily plays an essential role in the resistance of *A. baumannii* to different antibiotics. CraA is an efflux pump involved in the intrinsic chloramphenicol resistance of *A. baumannii* strains [191]. Ribbera and colleagues reported the involvement of *tet(A)* gene in the tetracycline resistance [192]. Recently, Foong and colleagues have shown that overexpression of *tet(A*) and *tet(G)* genes confers resistance to doxycycline, minocycline and tetracycline. It has also been observed that *tet(A)* acts additively with efflux pumps in the RND system, acting as a determinant of tigecycline resistance. *Tet(A)* gene is involved in the efflux of tigecycline into the periplasm, being subsequently eliminated by the AdeABC and AdeIJK pumps from the outer membrane. The synergistic mechanism of *tet(A)* gene with pumps from the RND family has an important role in the efflux of tigecycline in *A. baumannii* [193]. Other examples of efflux pumps from the MFS family are AmvA, which confers resistance to different classes of antibiotics, disinfectants and dyes [194] and AbaF, which is associated with fosfomycin resistance [195]. Recently, Perez-Varela and collaborators identified a new efflux pump from the MFS family, later called AbaQ, with role in virulence. Subsequent analyses have shown that AbaQ is the first pump in the MFS family involved in the outflow of quinolone-type antibiotics [196].

SMR is another efflux pump category described in *A. baumannii*, which includes AbeS, an efflux pump whose role was initially characterized by Srinivasan and coworkers, reporting its involvement in resistance to a several antibiotics and dyes, and subsequently, by Lytvynenko and colleagues, who analyzed the molecular basis of the multiple specificities of AbeS [197,198].

#### 3.2.2. Decreased Membrane Permeability

Decreasing the degree of membrane permeability may increase the AR. The pores of the outer membrane have an essential role in the resistance and virulence of *A. baumannii* strains, by mediating the transport of the molecules [199]. In *A. baumannii*, decreased membrane porin density (Omp22–23, Omp43, Omp44, Omp47, Omp33–36, Omp37 and CarO) is associated with increased carbapenem resistance [200,201,202,203,204,205,206,207,208,209]. Another described membrane porin involved in the pathogenesis of *A. baumannii* strains is OmpA. Smani and colleagues reported the association of this porin with aztreonam, chloramphenicol and nalidixic acid resistance [210] and recent studies have highlighted the role of OmpA in increasing the virulence [211], in lung infections, sepsis—and increased the mortality [212,213].

#### 3.2.3. Changing the Target Site

Alteration of the target site of the antibiotic is an essential mechanism of bacterial resistance. In general, this mechanism is based on random point mutations that have a minimal impact on bacterial cell homeostasis. Mutations can occur in any species and have recently been shown in *H. pylori* and *S. aureus* strains. The 23S ribosomal RNA mutations in *H. pylori* cause resistance to clarithromycin [214]. Studies have shown that point mutations in the same nucleotide sequence are associated with increased linezolid resistance [215]. Given the reports on the expansion of the resistance phenomenon, including against last-line antibiotics, we will only present the mutation-mediated resistance mechanisms of *A. baumannii* strains to the most common antibiotic classes used in the treatment of the infections.

One of the most discussed examples through point mutations is rifampin resistance (RIF). Rifampin blocks RNA polymerase activity. The region to which rifampin binds is a highly conserved enzymatic structure in the β subunit of RNA polymerase encoded by the *rpo*B gene. After the attachment to the binding site, the antibiotic molecule blocks the transcription by inhibiting nascent RNA [216]. It has been observed that increased resistance to rifampin is associated with the occurrence of point mutations in the *rpo*B gene, which result in various changes in the amino acid chain [217]. In *A. baumannii*, rifampin resistance can also be mediated by the *arr-2* gene encoding an ADP-ribosyltransferase, found within class 1 integrons.

Another example induced by point mutations is fluoroquinolones resistance. Fluoroquinolones act by inhibiting the DNA gyrase and topoisomerase IV enzymes encoded by gyrA and parC genes. In *A. baumannii*, the most common fluoroquinolone resistance mechanism is represented by the spontaneous mutations in the *gyrA*, *gyrB* and *parC* genes encoding gyrase and topoisomerase IV. The investigation of 56 *A. baumannii* strains harvested from 23 hospitals showed a strong association of fluoroquinolone resistance with triple mutations in the *gyrA, gyrB* and *parC* genes [218]. Ardebili et al. demonstrated the correlation of fluoroquinolones resistance with mutations in the DNA gyrase and topoisomerase IV encoding genes, after analyzing 55 isolates of *A. baumannii* [219], and more recently, in case of 23 strains investigated in Cairo [220]. Some clinical studies have shown that single *parC or gyr* mutations often lead to reduced susceptibility to fluoroquinolones, but without full resistance. In addition, additional target mutations will generate full clinical resistance, with high MICs breakpoints [221,222]. Other resistance mutations occur in regulatory genes that control the expression of outer membrane proteins and efflux pumps [221].

Fluoroquinolone resistance is also mediated by plasmid-mediated quinolone-resistance genes (PMQR), either through the QNR protein, a pentapeptide that protects target enzymes from antibiotic action [223] or through an aminoglycoside-modifying mutant enzyme [224].

Extensive use of colistin as last-resort treatment led to the development of colistin resistance. Regarding the colistin action, it is believed that colisting binding to lipopolysaccharides (LPS) in the outer membrane of Gram-negative bacteria causes changes in the structure of the phospholipid bilayer that leads to cell death by installing an osmotic imbalance [225]. Despite that, in 1970, it was replaced with other antibiotics due to side effects such as nephrotoxicity and neurotoxicity [226]. However, the severity of nosocomial infections forced the use of colistin as last-resort treatment [227]. Consequently, the extensive use of colistin caused the emergence of resistance, first observed in 1999 in the Czech Republic [228] and later globally. In *A. baumannii*, two mechanisms of colistin resistance have been described: i) modification of the lipid A from LPS by mutations in the PmrAB two-component system and ii) loss of LPS production capacity due to mutations in the *lpxA*, *lpxC* and *lpxD* genes. In 2009, Adams et al. analyzed the sequences of PmrAB components in *A. baumannii*-susceptible and resistant strains. They found mutations in the *pmrA* and *pmrB* genes in the case of resistant strains [229]. This finding led to the idea of the role of PmrAB component in regulating the colistin susceptibility of *A. baumannii,* by regulating the NaxD deacetylase transcription and the modification of lipid A by *β*-galactosamine deacetylase [230]. Mutational inactivation of the *lpxA*, *lpxC* and *lpxD* genes, which are involved in the LPS biosynthesis, results in the loss of antibacterial activity of colistin.

All these complex resistance mechanisms highlight the imperative need to analyze the AMR in *A. baumannii,* in order to respond to current challenges by developing innovative, practical therapeutic approaches.

## 4. Innovative Strategies for Treatment of *A. baumannii* Infections

Studies conducted in recent years highlight the unique involvement of *A. baumannii* strains in increasing the severity of nosocomial infections and, implicitly, their associated morbidity and mortality rates. Analysis of the resistance profiles of *A. baumannii* strains reveals the spread of the MDR phenomenon to most common antibiotics, including last-line ones [21,25]. As an alternative to this phenomenon, studies have initially focused on the approach of combined therapies such as minocycline/tigecycline [231,232], colistin/tigecycline [233], colistin/rifampin [234], polymyxin B-combined therapy [235]. All of these alternatives have only limited effects in case of nosocomial infection treatment, inevitably leading to a selective pressure that increases the resistance level of bacterial strains. Therefore, in the long run, combined antibiotic therapy may not be feasible in clinical settings. Given that *A. baumannii* strains are resistant to almost all antibiotics used [229], the research direction must be in line with the “post-antibiotic era”, emphasizing the development of innovative strategies to control MDRAB spreading. Next, we will present the most innovative therapies, such as new antimicrobial peptides, phage therapy, and CRISPR Cas system (Clustered regularly interspaced short palindromic repeats) developed to prevent the spread of MDRAB strains.

### 4.1. Antimicrobial Peptides (AMP)

Antimicrobial peptides may represent an alternative to antibiotics in the control of MDRAB strains spread. AMP is a class of compounds widespread in the living world as part of the innate immunity, acting as a primary barrier against infectious agents such as viruses, bacteria and fungi [236,237]. AMPs also play an essential role in regulating immune processes such as activating and recruiting immune system cells, angiogenesis and inflammation [238]. AMPs are amphipathic molecules with a positive electric charge, having a length of about 11–50 amino acid residues [238,239]. The main mechanisms of antimicrobial action of AMPs are the ability to cause cell membrane and cell wall damage, the inhibition of protein synthesis, nucleic acids and the induction of apoptosis and necrosis [240]. Due to these properties, AMPs have been considered an alternative to the use of antibiotics for limiting the spread and decreasing the infection rate and mortality control measures of nosocomial infections.

Cathelicidins are a group of AMPs detected in the immune system of some vertebrates that have in their structure two domains involved in antimicrobial activity [241]. One of the best-known cathelicidins is Cath-BF, isolated from the venous glands of the species *Bungatus fasciatus*. Starting from Cath-BF, several derived forms, such as ZY4 and Cath A, have been obtained and tested for antimicrobial activity. Other cathelicidins with antimicrobial activity, identified in the venous glands, are OH-CATH30, from the venom of king cobra and myrtoxin, from *Myrmecia pilosula* [242,243]. In vitro and in vivo studies have revealed the antimicrobial activity of these compounds, manifested by inhibition of planktonic and biofilm bacterial growth, eradication of persistent bacterial cells and inhibition of the inflammatory process [242,244,245]. However, despite the proven antimicrobial activity, further studies are needed to obtain information about expression sites and the influence of these peptides on microbial and host cells [241]. Compounds with similar activity have been identified in the venom of some scorpion species and tested against antibiotic-resistant bacteria. In the case of *Leiurus quinquestriatus*, a broad antimicrobial activity of the tested compounds was observed. However, there is not enough information regarding the interaction of these compounds with specific molecules of some microorganisms [246]. A high antimicrobial activity was observed for AMPs obtained from *Vespa affinis* (mastoparan) [247], *Capra hircus* (minibactenecins) [248], *Lucilla serricata* (sarcotoxin) [249] and from *Rana catesbeiana* (ranalexin and danalexin) [250]. However, despite the antimicrobial activity on *A. baumannii* strains of these compounds, further in vivo studies are needed to improve the pharmacokinetic properties for systemic administration, as well as to find solutions to avoid their degradation by proteases. Jakiewicz et al. studied the antimicrobial activity of eight peptides on *A. baumannii* strains. Among these, CAMEL and pexiganan showed potent antimicrobial and anti-biofilm activity. CAMEL is a hybrid AMP consisting of cecropin from *Hyalophora cecropia* and melittin from *Apis mellifera*. This study demonstrates the potential of these compounds to act against resistant strains [251]. Intense activity against biofilms have been observed in cecropins identified in *Musca domestica* [252], myxinidin isolated from *Myxine glutinosa* [253] and in the complex of AMPs (Fly larvae immune peptides) from *Calliphora vicina* [254]. Natural AMPs can be a starting point for the biosynthesis of AMPs with similar functions, being an attractive therapeutic option for the prevention and control of *A. baumannii* infections. Such examples of synthetic AMP are stapled AMP [255] and PNA (RXR) 4 XB, an antisense nucleic acid peptide compound [256] with intense bactericidal activity. However, the need for high doses to increase efficacy leads to the need for further in vivo studies to observe possible side effects. To be considered for therapy, AMPs must have a broad spectrum of action, high specificity and low cytotoxicity levels to mammalian cells [257]. The primary limitations that hinder the approval of systemic use of AMPs are sensitivity to enzymatic digestion and high toxicity, which is why most AMPs are applied topically and not orally or intravenously [258,259]. It has also been observed that certain physiological conditions, such as high concentrations of salts and serum components, can exert adverse effects on AMPs [260]. Compared to the conventional use of antibiotics, production costs for AMPs are much higher, which is why research is moving towards peptides as short as possible with stable properties [261]. Some studies reveal the appearance of AMPs resistant strains [262], the best-known example being *A. baumannii* resistance to colistin [21,25]. Currently, research is aimed at developing technologies to improve the efficiency of AMPs in vivo, especially in terms of increasing the specificity against the infectious agents, decreasing cytotoxicity to mammalian cells, increasing stability and lowering production costs. The newest AMPs studied to elucidate their therapeutic efficacy against *A. baumannii* strains are summarized in Table 3.

### 4.2. Bacteriophages Therapy

Bacteriophages are viral parasites able to infect bacteria by recognizing surface receptors, injecting their genetic material into the host and replicating using the host cellular machinery [281]. Phages exhibit ecological and genetic effects on bacteria at the population level, and these effects can impact plasmid stability [282,283]. Phages may enhance the persistence of ARGs as an adaptation strategy to restrictive environmental conditions, e.g., wastewater aggressively treated using UV, temperature or pH. However, genetically modified phages could be used to increase antibiotic susceptibility of resistant strains. The alarming increase in the resistance rates has also led to the revival of phage therapy to increase the susceptibility level of bacteria by eliminating resistance and virulence markers [284]. Phages do not exert adverse effects on the patient’s microbiome and have a high degree of selectivity and specificity for pathogens [285]. In addition, research has shown that phage therapy has a high potential to represent an effective and safe treatment against MDRAB strains [286,287]. A high number of experiments were performed both in vitro and in vivo, the main results being summarized in Table 4.

Bacteriophage therapy represents a promising tool in fighting MDR *A. baumannii* strains. Analyzation of the data summarized in Table 4 highlights that both in vitro and in vivo studies demonstrate the high efficiency, increasing the survival rate of organisms infected with *A. baumannii* strains. Based on the results obtained on animal models in the last ten years, numerous studies have focused on understanding the effectiveness of this therapy against chronic infections in the hospital units, as revealed by different clinical trials [284,301,302,303,304]. Schooley et al. have used phagotherapy in a patient with necrotic pancreatitis caused by an MDRAB strain [305].

Contrary to these studies, there are other reports of the inefficiency of phages in treating bacterial infections [306,307], which suggests that the clinical use of phages requires standardization. One of the most significant challenges in phage therapy is the resistance of bacterial strains to phage action [308,309,310]. In bacterial communities, resistance is a dynamic process when the antibacterial agent is biologic, as is the case with phages. On the other hand, phages exert a selective competition on bacteria. This two-way interaction causes a co-evolution that results in bacteria acquiring resistance mechanisms that can block the cycle of lytic infection [308,309]. Bacteria can acquire resistance to phages following cellular surface changes represented by point mutations in phage binding receptors [311]. Another mechanism of resistance is the outer membrane vesicles to which phages can bind due to surface structures, similar to those of parental cells. Binding of phages to these vesicles during invasion decreases the likelihood of cell infection [308]. In addition, a significant impact has the restriction–modification systems, the most common defense mechanisms in bacteria that can degrade foreign DNA, including double-stranded DNA phages [312]. Another concern in the use of phage therapy is the lack of standardization of phage preparation methods. An incomplete purification of host bacterial phages can lead to an unwanted transfer of bacterial toxins such as endotoxins or exotoxins [313]. Particular attention should be paid to combination therapy with phages and lysins. Once the dose needed to increase antimicrobial action has been determined, the mechanisms of action and elimination from the body must be established [314]. Stimulation by the phage of the immune response and adaptive immune systems, as well as their presence in the bloodstream, may influence the effectiveness of phage therapy [313]. Van Belleghen et al. observed the process of phages’ opsonization by binding to the surface of invasive bacteria that can lead to neutralization of phages by the secondary adaptive immune response. Thus, the recognition of circulating phages can lead to their elimination. In addition, phages can be detected in the systemic circulation by tissue proteases or the reticuloendothelial system and delivered to the liver and spleen where degradation occurs [313].

Further studies are required to understand phage biology clearly and to better control clinical trials to standardize phagotherapy.

### 4.3. CRISPR System-a New Approach in the “Post-Antibiotic Era”?

As mentioned previously, the current strategies used to combat MDRAB infections have many limitations. In the recent years, one of the most attractive alternatives to combat bacterial resistance is the use of CRISPR (clustered regularly interspaced short palindromic repeat) system described for the first time in 1987, by Ishino et al. [315]. CRISPR/Cas is an immune defense system encountered in bacteria able to recognize and degrade foreign nucleic acids through associated caspases.

**Figure 1:**
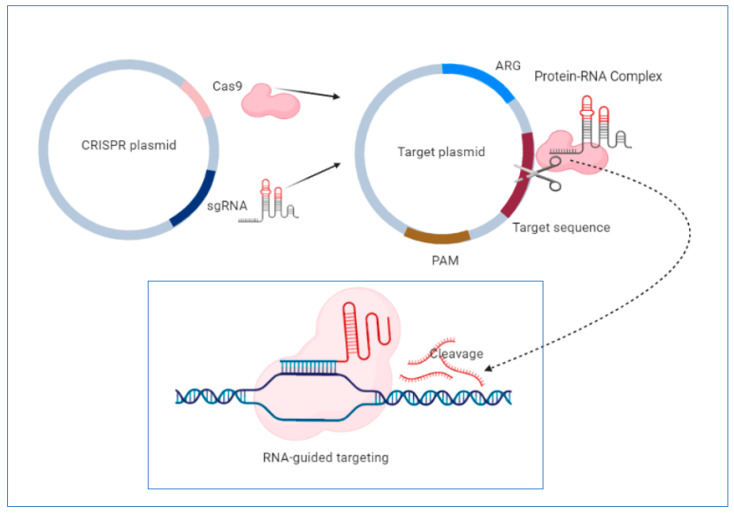
Schematic representation of clustered regularly interspaced short palindromic repeat (CRISPR)-based targeting of mobile genetic elements (MGEs). This system contains the cas9 nuclease, sgRNA transcript and other structural elements. In the first stage, sgRNA forms a complex with Cas 9 nuclease. The sgRNA transcript guide cas9 nuclease to introduce double-stranded breaks at the ends of the target DNA, leading to cleavage. Direct target recognition is achieved by recognizing protospacer adjacent motifs (PAM), short DNA sequences that are not found in CRISPR loci, so there is no risk of self-degradation. This system can be used to edit the genome of several antibiotic-resistant bacterial strains, leading to the removal of resistance determinants. Figure created with https://biorender.com/.

One of the most significant advantages of this system is its high specificity, based on the existence of short, repetitive sequences in CRISPR loci separated from each other by single sequences of 26–72 pairs of lengths derived from MGEs such as plasmids or transposons [316]. The defense mechanism against exogenous genetic elements is accomplished in three stages: acquisition, expression and interference [317]. The acquisition stage involves the insertion into repetitive loci of the host chromosome of single sequences (spacers) derived from MGEs, separated by repetitive sequences. The expression consists of transcribing the complex formed of repetitive and spacer sequences into a single RNA transcript that will be further processed by caspases in short CRISPR RNAs. Caspases, including ribonucleases, are a family of protease enzymes, playing essential roles in programmed cell death [318]. In the interference phase, foreign nucleic acids are identified based on complementarity with CRISPR RNAs, and their degradation is accomplished by caspases [319]. Discrimination between self and non-self is accomplished through sequences from the foreign nucleic acid called protospacers. These sequences are placed between some sequence motifs called PAMs (adjacent protospacer motif). Direct target recognition is achieved only by identifying these sequence motifs not stored in CRISPR loci, so there is no risk of degradation of its nucleic acid [320] (Figure 1).

In *A. baumannii*, two types of CRISPR systems were identified within MGEs containing different spacer sequences. In addition, the distribution of some analyzed isolates in different clusters was observed, suggesting that this system was acquired by HGT throughout evolution [321,322]. Karah et al. analyzed 76 *A. baumannii* isolates to study the I-Fb subtype of the CRISPR system. Forty types of CRISPR sequences were revealed, two being found mostly in 35.52% of the analyzed isolates, suggesting the existence of two primary clones. The spacer sequences are arranged in chronological order of their inclusion from the invading nucleic acids so that the old ones are positioned at the end. This temporal positioning of the sequences allows the use of the CRISPR system for the micro- and macroepidemiological classification of clinical isolates [323].

These results have opened new perspectives regarding the possibility of using the CRISPR system for subtyping *A. baumannii* strains [320,324,325,326,327,328,329,330,331,332]. Wang et al. developed a CRISPR platform that allows rapid genomic editing by introducing deletions, insertions, and point mutations to analyze the mechanisms involved in oxidative stress (OxyR) in *A. baumannii* strains. For the introduction of deletions, the authors constructed a CRISPR plasmid in which they incorporated the CRISPR elements from *Streptococcus pyogenes*. To repair double-strand breaks, they used RecA recombinase from *A. baumannii*. The introduced mutations in the oxyR gene and its deletion, lead to a high susceptibility level of *A. baumannii* strains to oxidative stress, demonstrating the importance of this gene as a central transcriptional regulator of the response to oxidative stress [333]. Mangas et al., conducted an in silico study to analyze the pan-genome of 2500 *A. baumannii* strains. Depending on the number of shared genes, the authors observed that genomes are divided into two broad groups. The group of strains with a lower number of genes shows the sequences and genes characteristic of the CRISPR system, genes specific to the toxin–antitoxin system and genes involved in the biofilm formation, which is why it is considered that the CRISPR system may have an essential role in virulence. Unlike the strains from group two, positive for a high number of plasmids, the strains from the first group contain mainly genes involved in regulating the elements of the CRISPR system. This finding led to the idea that the CRISPR system may be involved in the restriction of the plasmid entry into bacterial cells [334]. Karlapudi et al. conducted an in silico study to understand the role of the AbaI gene in biofilm formation in *A. baumannii*. For this, they used a series of genetic editing tools to create AbaI gene knockouts. The analyzed tools (CHOCHOP, CCTop, E-CRISP, CRISPR Direct, Off-Spotter, Crispr-era) can provide information about the target sequences, specific primers, existing mutations, the location of the target sequence in order to perform the knockout, as well as the necessary sgRNA sequences for performing genomic editing experiments [335]. The information obtained from the in silico genomic experiments demonstrates the need to improve genetic editing tools. In addition, further studies should consider the construction of sgRNA with a custom design, depending on the diversity of cell types.

Despite the notable results obtained from using the CRISPR system to combat antibiotic resistance, however, controlling bacterial populations using this strategy has some limitations. First of all, the appearance of mutations outside the target represents a significant limitation of the CRISPR system. In addition to cleaving target sequences, the CRISPR system can act on identical or homologous DNA sequences, leading to mutations in unwanted sites, called off-target mutations. Mutations outside the target can lead to cell death or transformation, which is why it is recommended to select target sites at which as few mutations can occur outside the target [336].

Another challenge in using the CRISPR system in controlling bacterial populations is the need for PAM sequences that are involved in differentiating between self and non-self. There is a limitation of the number of target sites due to the need for these sequences. Because the CRISPR system requires specific PAM sequences to function, their genetic engineering processing can eliminate this limitation [336].

Another major limitation in using the CRISPR system in the control of bacterial populations is represented by the delivery of the protein–RNA complex through the bacterial membrane. There is the problem of delivering the CRISPR system in the case of both Gram-negative and Gram-positive bacteria. It has been observed that the techniques used to encapsulate the gRNA-protein complex have a significant impact on loading and packaging efficiency, thus limiting their practical use [337,338]. Consequently, studies have focused on the use of phages as a vehicle for the delivery of the CRISPR system at the target level. This strategy involves encapsulating the CRISPR system in the capsid of inert phages [326,327]. Starting from phages as a delivery vehicle of the CRISPR system in vitro, the problem of controlling bacterial populations using the CRISPR system in vivo was raised. Starting from the fact that oral administration of phages for targeting bacteria in the intestinal tract was used successfully [331], one strategy is to use phages as a vehicle for delivering the CRISPR system in the intestinal microbiota, for eliminating the ARGs. However, it is required to have a collection of phages specially designed to target ARGs, to establish the optimal concentration required and to know the several barriers that occur in vivo, such as inactivation of bacteriophages by gastric acid, neutralization of phages by the spleen and the immune system [332].

## 5. Discussion

The mentioned studies highlighted the particular involvement of *A. baumannii* strains in different infections types, as well as the need to implement appropriate infection control measures to limit the spread and decrease the infection rate and mortality. Data summarized in Table 1 and Table 2, emphasize that in the great majority of *A. baumannii* isolates, the primary mechanism of AR is represented by the production of diverse *β*-lactamases, from all four Ambler classes, both chromosomal and plasmidial. Among these, CHLDs seems to be mostly related to the occurrence of MDR phenotypes in *A. baumannii*. Although colistin was initially one of the leading antibiotics in the treatment of nosocomial infections caused by *A. baumannii*, a gradual increase of colistin resistance in clinical isolates, reaching even 100% in some studies has been revealed [19,21,25]. The origin of the clinical isolates demonstrates the wide dissemination of infections, the *A. baumannii* resistant strains being isolated mostly from tracheal aspirates, tracheal secretions, burn wounds, blood, urine or cerebrospinal fluid (Table 1). In addition, most of the patients were admitted to ICUs, which highlights the severity of infections caused by *A. baumannii*, which can cause increased morbidity, hospitalization length and costs, as well as mortality.

To date, a significant challenge in the clinic is to develop methods to establish the appropriate MIC values needed to increase treatment efficiency. Most laboratories are not able to determine MIC values accurately and reproducibly enough and to eliminate variations. One cause of variations in MIC values is the existence of several strategies and methods to determine these values. Terwee et al. studied the differences obtained in MIC values following the use of several methods that fall into two general categories: “anchor” methods and distribution-based methods. Anchor methods use an external criterion to establish a significant change (patient opinion), and distribution-based methods use statistical data to determine the MIC value. In the case of applying the two strategies, Terwee et al. observed significant differences in MIC values [339]. These significant differences could be closely related to population characteristics such as age, the severity of the condition and treatment and the method used. In this situation, several factors that contribute to variability make it difficult for clinicians to establish a single MIC value or at least a range of values as small as possible [339].The semiautomatic susceptibility detectors often provide truncated MIC values. The same problem exists with gradient tests, such as the E-test, which may omit a certain percentage of resistant strains, leading to treatment failure in the clinic [340]. Even if the susceptibility tests are performed correctly, variations may occur due to discontinuous results reported at a specific interval, usually at a 2-fold scalar dilution. When variations occur, MIC values may exceed these intervals, leading to incorrect doses of antibiotics, which may be harmful to the patient [341].

For this reason, it is imperative to standardize the methodology for identifying MIC values in microbiology laboratories. The standardization process is essential for prescribing a correct treatment, controlling severe infections, and for stopping the expansion of the resistance phenomenon.

Moreover, the in vitro susceptibility tests used for prescribing a treatment do not provide information about the bacteriostatic or bactericidal activity of an antibiotic [342].

Bacteriostatic activity refers to the inhibition of bacterial growth, and bactericidal activity refers to the killing of bacteria. In reality, there is no particular antibiotic that either inhibit or kill bacteria. In patients with inflammatory and immunocompromised diseases, it is critical to identify the minimum bactericidal concentration (MBC). MBC is the lowest concentration of antibiotic that kills bacteria, reducing bacterial colonies by up to 99% [343]. Whether we are talking about the curing strategy, based on the identification of MIC values or the identification of MBC in order to eradicate bacteria, there are advantages and disadvantages in both treatment strategies. In the case of diseases such as endocarditis [344], meningitis [345], osteomyelitis [346] and neutropenia [347], the use of bactericidal action is recommended. High bacterial concentrations, the presence of dormant bacteria with high resistance, low immune competence of the body and low ability to penetrate the antibiotic are some factors that lead to the need to use agents with bactericidal activity to achieve complete sterilization of the infectious outbreak., The use of bactericidal agents is recommended in some clinical situations, but there are disadvantages to bactericidal action. The rapid action of bactericidal agents can have adverse clinical consequences, such as the discharge of endotoxins following bacterial lysis. Rapid eradication of bacteria can lead to cell wall fragments and pneumolysins, which can exacerbate the immune response, the release of prostaglandins and high mortality rates in meningitis [348]. Therefore, the risk of a significant inflammatory reaction due to bacterial lysis must be considered.

The heterogeneity and increased efficiency of the resistance mechanisms of *A. baumannii* strains against almost all existing antibiotics threatens with the transition to the “post-antibiotic era”, indicating the acute need to search for new therapeutic approaches. One of the challenges that hinder the success of the treatment of severe infections is the emergence of the phenomenon of heteroresistance. Heteroresistance occurs when subpopulations of isogenic bacteria exhibit lower susceptibility than the general population [349]. In *A. baumannii*, heteroresistance has been reported in antibiotics such as aminoglycosides, tobramycin, gentamicin and imipenem [350,351], but also in other antimicrobial agents such as AMPs [352]. Currently, the biggest threat is the reporting of colistin heteroresistance, which implies the existence of resistant subpopulations in a susceptible isolate (MIC ≤ 2 mg/L) by in vitro susceptibility tests [353]. In the case of severe nosocomial infections produced by heteroresistant strains in the clinic, colistin treatment may cause resistance expansion and, thus, treatment failure [354]. For detection of the phenomenon of heteroresistance, various methods are used, such as BMD (broth microdilution), E-test or PAP (population analysis profile) [353]. The use of appropriate susceptibility tests to identify heterogeneous subpopulations is essential for the success of clinical treatment. The study by Caglan et al. highlighted differences between the results obtained after the application of BMD and E-test. Using E-test, the resistance to colistin was 4.2%, while by the BMD method, a percentage of 25.8% was obtained, analyzing the same isolates. Therefore, in the case of the E-test, a large part of the resistant strains has not been identified, which is a significant mistake in the clinic [340]. Thus, clinicians should keep in mind that although the use of gradient tests is more comfortable, the results can be confusing and can negatively influence patients’ treatment.

Another problem that clinicians need to consider is the emergence of heteroresistance in patients who do not have a history of colistin treatment. However, it is more common in patients who have received treatment [355]. The emergence of heteroresistance to a range of antibiotics, including last-line antibiotics, significantly impedes the management of severe nosocomial infections caused by MDRAB strains and requires increased clinical attention to identify resistant subpopulations.

## 6. Conclusions

Different MDR microorganisms, among which *A. baumannii* are opportunistic pathogens, able to compete in new environments where previously only commensals or non-pathogenic microorganisms existed. The survival and persistence in nosocomial environments characterized by high antimicrobial pressure have led to the emergence of *A. baumannii* as a key pathogen, whereas a few decades ago, it caused practically no disease. The incidence of MDR and virulent clones of *A. baumannii* is also increasing worldwide, at least in these specific settings.

One of the clinic’s difficult challenges is establishing the correct MIC values based on which to prescribe a correct treatment. The existence of numerous factors that influence the MIC values such as the lack of standardization of the methodology makes the success of the therapy difficult. Increased attention should be paid to factors that may influence MIC values such as patient characteristics (age, disease severity) and methods applied. In addition, identifying MIC values and MBC values can provide clinicians with additional information about the antibiotics needed to prescribe the most appropriate treatment.

The emergence of heteroresistance in some bacterial subpopulations is another challenge in the management of infections caused by *A. baumannii*.

The enormous adaptability of *A. baumannii*, as well as the very diverse mechanisms for the acquisition and transfer of AR determinants, contribute to the inefficiency of most current therapeutic strategies, determining the transition to the “post-antibiotic era” and highlighting the necessity to develop new therapeutic approaches. The latest strategies include obtaining the use of AMPs, bacteriophage therapy and CRISPR technology. Although experiments have shown the potential of these strategies in combating MDRAB, there are several challenges, such as the narrow spectrum of action, low specificity, high cytotoxicity, sensitivity to enzymatic degradation and bacterial resistance. These limitations must be addressed in future studies to develop efficient strategies for the optimal management of MDRAB infections.

## Figures and Tables

**Table 1:** Antibiotic resistance (AR) profiles of A. baumannii *.

*A. baumannii* Isolates Number	AR Profile	Minimal Inhibitory Concentration (MIC) Range	Resistant Isolates (%)	Hospital Wards/Origin	References
164	GEN, AMK	256–≥1024 mg/L	– – 32.1%–83.8%	– – Endotracheal aspirates, tracheal secretion, wound tampon, pus, sputum, catheters/ ICUs (Intensive Units Care)	[14]
	Cephalosporins, Carbapenems	– U ^a^			
44	PIP, CTX, CAZ/ATM, IMP	0.5–256 mg/L	79.5%	U	[15]
121	CIP, AMK, AMP–SUL, ATM, CTX, GEN, NET, PIP, TIM, TOB, IMP	0.5–256 mg/L	0%–92.6%	Bronchial cultures, burns, blood culture, catheters/ ICUs	[16]
375	AMP, PIP, PTZ, CAZ, CTX, IMP, MEM, GEN, AMK, CIP	0.5–>256 mg/L	0%–100%	Sputum, wounds/ ICUs	[17]
23	CAZ, CTX, FEP, PIP, TZP ^b^, ATM, IMP, MEM, CIP, AMK, GEN, SXT	0.75–>256 (µg mL^−1^) ^b^	100%	U	[32]
72	IMP, MEM, FEP, CAZ, SUL, CFP–SUL, PIP, TZP, ATM, CIP, AMK, TIG	4–≥512 mg/L	77.8%	ICUs –	[36]
100	IMP, MEM, CAZ, CST, TET, TIG, AMP–SUL	0.25–256 (µg/mL) ^b^	0%–100%	Sputum, wounds, blood culture, urine, fluids, hemodialysis catheters; ACH (Acute Care Hospital)	[37]
204	TIC, TIM, CAZ, FEP, ATM, IMP, TOB, KM, GEN, AMK, PFX, LVX, OFX, CST, TET, FOS	U	92% for β-lactams; 47% for IMP	Puncture, pus, blood culture samples/ICUs	[41]
20	TIC, CAZ, IMP, MEM	4–>256 µg/mL	90%–100%	Tracheal aspirate, bile, urine, burns, respiratory tract, blood culture, sputum/ICUs	[38]
100	PIP, AMP/SUL, CIP, AMK, IMP, CTX, FEP, CRO, TET, GEN	U	45%–100%	Burns, sputum, tracheal secretion, pleural fluid, blood culture, urine, cerebrospinal fluid/ ICU	[42]
834	AMC, CIP, GEN, AMK, CFP–SUL, IMP, MEM	U	71.7%–96.8%	Tracheal aspirate, blood culture, urine, wounds/ICU	[43]
15	IMP, MEM, GEN, GEN/NMP, CIP, CIP/NMP, CAZ	0.25–≥256 mg/L	U	Gastroenterology	[44]
155	CIP, FEP, TZP, CAZ, IMP, MEM, AMP/SUL	U	93.5%–94.4%	Blood respiratory tract secretions, catheter, urine	[39]
65	AMK, AMP/SUL, FEP, CST, SXT, DOX, IMP, LVX, MEM, MIN, SUL, TIG, TOB	0.125–256 mg/L	1.5%–96.9%	Respiratory tract secretions	[24]
59	TIC, PIP, AMP/SUL, TZP, CAZ, FEP, IMP, MEM, CST, GEN, TOB, AMK, MIN, CIP, LVX, SXT	U	10.17%–96.61%	CCNU (critical care nursing unit); UPU (emergency unit); RE (resuscitation); IM (internal medicine); NE (Nephrology); HO (hematology/Oncology); TC (thoracic cardiology); GS (general surgery); NEO (neonatology)	[25]
15	TIC, TIM, PIP, TZP, CAZ, CTX, FEP, MEM, NAL, LVX, CIP, GEN, TOB, AMK, SXT, IMP	U (exception MEM and IMP–32 mg/L)	40%–100%	Burns, pleural fluid, urine, bronchoalveolar lavage, pus, blood culture	[26]
40	CAZ IMP GEN	U	92.5% 85% 70%	ICUs, IM, GS	[27]
41	AMP/SUL, TZP, CAZ, CRO, FEP, IMP, MEM, GEN, CIP, TIG, AMK	4–≥128 mg/L	34.1%–100%	Tracheal aspirate, burns, urine, blood, exudate/ICU	[28]
84	GEN, AMK, CRO, FEP, CIP, LVX, CAZ, IMP, MEM, PMB, CST, AMP, TET, TIG, ATM	U	0%–100%	Wounds	[30]
35	Class III Cephalosporins Class II Cephalosporins Carbapenems Aminoglycosides Fluoroquinolones	U	>90% 65% >90% 60% 90%	Tracheal secretion, urine, cerebrospinal fluid/ICU	[31]
8	CAZ, FEP, CTX, CRO, IMP MEM, TZP, AMP/SUL, GEN, CIP, TIG, CST	1–>128 mg/L	71.4%–100%	Blood cultures, urine samples, aspirate sputum, bronchoalveolar lavage fluid, wound swab, pus	[21]
41	Aminoglycosides Β-lactams Quinolones Tetracyclines	U	95%	Tracheal aspirate, peritoneal fluid, bronchial lavage/ ICU; UPU; NICU (Neonatal Intensive Care Unit)	[22]

* Percentage = %; GEN = gentamicin; AMK = amikacin; PIP = piperacillin; CTX = cefotaxime; CAZ/ATM=ceftazidime/aztreonam; IMP = imipenem; CIP = ciprofloxacin; AMP–SUL = ampicillin-sulbactam; ATM = aztreonam; NET = netilmicin; TIM = ticarcillin/clavulanic acid; TOB= tobramycin; AMP = ampicillin; CAZ= ceftazidime; MEM = meropenem; FEP = cefepime; TZP = piperacillin/tazobactam; SXT = cotrimoxazole; SUL = sulbactam; CFP–SUL = cefoperazone/sulbactam; TIG = tigecycline; CST = colistin; TET = tetracycline; TIC = ticarcillin; KM = kanamycin; PFX =pefloxacin; LVX = levofloxacin; OFX = ofloxacin; FOS = fosfomycin; CRO = ceftriaxone; GEN/NMP = gentamicin/ 1-(1-naphtylmethyl)-piperazine; DOX = doxycycline; MIN = minocycline; NAL = nalidixic acid; PMB = polymyxin; ^a^ U = unknown; ^b^ = according to CSLI criteria.

**Table 3:** Antimicrobial Peptides (AMPs) with antimicrobial activity against *A. baumannii*.

Organism	AMP	Type of Study	Animal Model	Main Results	References
NA	ZY4 cathelicidin-BF-15 derived	in vitro; in vivo	mouse septicemia infection model	Antibacterial activity in plasma; biofilm inhibition; kills persister cells; inhibition of infection and inflammation in vivo	[244]
NA	epsilon-poly L-lysine (EPL)-catechol	in vitro; in vivo	mouse burn wounds infection model	Reducing bacterial burden in vivo	[263]
*Vespa affinis*	mastoparan-AF	in vitro	NA	Potent antimicrobial activity	[247]
NA	chex1-Arg20 amide (ARV-1502)	in vivo	Mouse infection model	Reduction of bacterial load	[264]
NA	*α*-helical -26 AMP residues	in vitro	NA	Great antimicrobial activity	[265]
*Delftia* spp.	delfibactin A	in vitro	NA	Great inhibitory effects	[266]
*Capra hircus*	mini-ChBac7.5Nα mini-ChBac7.5Nβ	in vitro	NA	Significant antimicrobial activity; induce membrane damages;	[248]
Hybrid striped bass *Morone saxatilis* × *M. chrysops*	I16 K-piscidine-1 analog	in vitro; in vivo	Sepsis mouse model	Strong bactericidal activity; high survival rate of infected mice;	[267]
*Musca domestica*	cecropin-4	in vitro	NA	Great bactericidal activity against MRAB and PRAB; inhibits biofilm formation	[252]
NA	Ω17 and Ω76 family peptides	in vitro; in vivo	Mouse peritoneal infection model	Disrupt bacterial membranes; induce small-molecule leakage; rapid bactericidal activity;	[268]
NA	ceragenins (AMP synthetic mimics)	in vitro	NA	Antibiofilm activity; inhibitory effects	[269]
*Medicago truncatula*	nodule-specific cysteine-rich (NCR) peptide and its derivatives	in vitro	NA	Potent killer of pathogenic bacteria	[270]
NA	TAT-RasGAP_317−326_ anticancer peptide	in vitro; in vivo	Mousel model of lethal peritonitis	Growth inhibition effects; broad-spectrum antimicrobial activity; great efficacy in vivo	[271]
NA	WLBU2-cationic amphipathic peptide	in vitro	NA	Eradicating bacterial biofilms;	[272]
*Myxine glutinosa* L.	myxinidin 2; myxinidin 3	in vitro, in vivo	Mouse skin wounds infection model	Antibiofilm activity; anti-inflammatory activity; enhance wound healing;	[253]
Hepatitis B virus	D-150–177C, HBcARD derivative peptide	in vivo	Mouse sepsis infection model	Strong bactericidal activity; 90% of mice protected from death;	[273]
*Pisum sativum*	nuripep 1653	in vitro	NA	Significant antimicrobial activity;	[274]
*Cimex lectularius* (bedbug)	CL defensin	in vitro	NA	Inducing membrane depolarization and pore forming; bactericidal action	[275]
*Bungarus fasciatus*	cathelicidin—BF derivate (Cath-A)	in vitro	NA	Bacterial growth inhibition	[245]
*Lucilia sericata*	LS-sarcotoxin and LS-stomoxyn	in vitro; in vivo	Mouse model infection	Strong activity against GRAM-NEGATIVE;	[249]
*Leiurus quinquestriatus*	venom cocktail proteins	in vitro	NA	Broad-spectrum antimicrobial activity; growth inhibition;	[246]
*Myrmecia pilosula*	Δ-Myrtoxin-Mp1a (Mp1a) heterodimeric peptide	in vitro; in vivo	Mouse model	Antibacterial activity; significant potency; nociceptive pain upon injection into mice	[243]
NA	glatiramer acetate	in vitro	NA	Efficient killing of clinical isolates	[276]
King cobra	OH-CATH30 D-OH-CATH30	in vitro; in vivo	Mouse model	Strong inhibition activity; low toxicity, great immunogenicity;	[242]
NA	stapled AMP Mag(*i*+4)1,15(A9 K, B21A, N22 K, S23 K)	in vitro; in vivo	Mouse peritonitis sepsis model	Great bactericidal activity; 88% of mice cured after intraperitoneal injection;	[255]
*Viola odorata*	Cy02 (cyclotide)	in vitro	NA	Strong bactericidal action	[277]
*P. aeruginosa* bacteriophage	artilysin 175	in vitro	NA	High, rapid and broad antibacterial activity against MRAB	[278]
*Calliphora vicina*	FLIP 7	in vitro	NA	Antibiofilm activity	[254]
Camel (colostrum milk)	lactoperoxidase lactoferrin	in vitro; in vivo	Acute pneumonia mouse model	Major inhibition effects; significant clearance of *A. baumannii* in lung and blood culture;	[279]
*Rana catesbeiana*	ranalexin danalexin	in vitro	NA	Strong antimicrobial activity	[250]
NA	PNA (RXR)_4_ XB	in vitro; in vivo	*Galleria mellonella* sepsis model	Excellent bactericidal activity in vitro; high dose of PNA conjugate required in sepsis model	[256]
NA	protegrin-1	in vitro	NA	Good activity against MRAB; no antibiofilm activity;	[280]
NA	aurein 1.2, CAMEL, citropin 1.1., LL-37, omiganan, r-omiganan, pexiganan and temporin A	in vitro	NA	CAMEL and pexiganan displayed the highest antibacterial activity	[251]

NA, not applicable; PRAB, polymyxin-resistant *A. baumannii*; HBcARD, human hepatitis B virus core protein arginine-rich domain; FLIP 7, fly larvae immune peptides 7; PNA (RXR)_4_ XB, peptide nucleic acid conjugated with cell-penetrating peptide.

**Table 4:** Bacteriophages therapy against *A. baumannii* strains.

Phages	Family	Isolation Source	Type of Study	Number of Tested Strains	% of Susceptible Strains	Animal Model Application	References
økm18p	*Corticoviridae*	hospital sewage	in vitro	34 MDR, 16 of those XDRAB	44.1%	NA	[288]
Acibel004	*Myoviridae*	wastewater sample	in vitro	34 MDR	82.3%	NA	[289]
Acibel007	*Podoviridae*	wastewater sample	in vitro	34 MDR	82.3%	NA	
IsfAB78	*Myoviridae*	water sample	in vitro	43 MDR	27.9%	NA	[290]
IsfAB39	*Podoviridae*	water sample	in vitro	43 MDR	25.5%	NA	
vB_AbaS_Loki	*Siphoviridae*	sludge	in vitro	34	5.8%	NA	[291]
Petty phage	*Podoviridae*	sewage	in vitro	40, 25 of those MDR	10%	NA	[292]
SH-Ab 15599	*Myoviridae*	sewage	in vitro	48 CRAB	27%	NA	[293]
SH-Ab15708	*Myoviridae*	sewage	in vitro	48 CRAB	29.1%	NA	
SH-Ab15497	*Siphoviridae*	sewage	in vitro	48 CRAB	29.1%	NA	
SH-Ab15519	*Podoviridae*	sewage	in vivo	48 CRAB	16.6%	Mouse model—lung infection; 90% survival rate	
vBGEC_AbM-G7was (phiG7)	*Myoviridae*	sewage	in vivo	200	68%	Rats wound model; 100% survival rate	[294]
Abp1	*Moraxelaceae*	sewage	in vitro	20	NA	Hella cells infection protection assay; 100% protection and survival rate of Hella cells.	[295]
			in vivo	20		Mouse local and systemic infection model; 100% survival rate.	
PB AB08	*Myoviridae*	Bacteriophage Bank of Korea	in vivo	14 MDR	35.7%	Mice model—intranasal phage cocktail; 35% survival rate	[296]
PBAB25	*Myoviridae*	Bacteriophage Bank of Korea	in vivo	14 MDR	7.1%	Mice model—ntranasal phage cocktail; 35% survival rate.	
WCHABP1	*Myoviridae*	hospital sewage	in vivo	2 CRAB	NA	*Galleria mellonela* infection model; 75% survival rate after phage administration	[297]
WCHABP12	*Myoviridae*	hospital sewage	in vivo		NA		
PD-6A3	*Podoviridae*	sewage	in vivo	552 MDR	32.4%	Sepsis mouse model; intraperitoneal administration; endolysin therapy, endolysin + phage therapy, phage therapy and phage cocktail; 70%, 70%, 60% and 50% survival rate.	[298]
Bϕ-R2096	*Myoviridae*	hospital sewage	in vivo	20 CRAB	NA	Galleria mellonella infection model; 80% and 50% survival rate at 96 and 48 h.	[299]
			in vivo		NA	Mouse model acute pneumonia; 100%, 60% and 30% survival rate at day 12, with MOI 10, 1 and 0.1	
AB3P1	NA	sewage, farm soil, feces of sheep, chicken litter, swab for surgical lounge.	in vivo	23	78.2%	Mice model; intraperitoneal administration of AB3 phages; 100% survival rate;	[300]
AB3P2	NA		in vivo				
AB3P3	NA		in vivo				
AB3P4	NA		in vivo				

NA, not applicable; MOI = multiplicity of infection.
